# REACH – an overview

**DOI:** 10.2478/v10102-009-0007-1

**Published:** 2009-06

**Authors:** Rostislav Čihák

**Affiliations:** Výzkumný ústav organických syntéz a.s., Centre for Ecology, Toxicology and Analytics, Rybitví, Czech Republic

**Keywords:** REACH, chemicals, testing, alternatives

## Abstract

An outline of the new European policy in the management of chemicals is given. This new system is known under the acronym REACH which stands for Registration, Evaluation and Authorisation of Chemicals. The background, objectives, requirements and the operation of REACH are briefly explained.

## Introduction

It was before eight years when first outlook of the new EU chemicals policy and regulation on chemical substances has became in recognition. The first concept of it was published in 2001 as so called White Book (Strategy for a Future Chemicals Policy).

After long term, difficult and demanding discussions among stakeholders (industry, regulators, non-governmental organizations) the new regulation dealing with the management of chemicals in the European Union entered into force under the acronym of REACH, which stands for Registration, Evaluation and Authorisation of Chemicals (Regulation EC No. 1907/2006).

The prolonged law making process has eventually come to an end, after an enormous amount of debate and consultation of interested parties and parliament members, culminating in final agreement by the European Parliament on 13 December 2006. The Regulation comes into force legally on 1 June 2007, but the scheme begins operating from 1 June 2008, to give time for the European Chemicals Agency (ECHA) to be set up in Helsinki and become operational. Also work on the REACH Implementation Projects continued and the development of the necessary guidance to industry and regulators was finished (now available at ECHA web site as Guidance Documents on REACH processes and methods, to be used by industry and authorities.)

## Background

In the beginning of the process it was generally accepted that the current EU chemical control measures result in too great a disparity between new and existing substances, with the high cost of new substance notification stifling innovation. Furthermore, existing substances account for over 99% by volume of chemicals in commerce, but are poorly assessed and controlled in comparison to new substances.

The former EC legislative framework for chemical substances was a patchwork of many different Directives and Regulations which has developed historically. There were different rules for “existing” and “new” chemicals. However, this system did not produce sufficient information about the effects of the majority of existing chemicals on human health and the environment. The identification and assessment of risks – covering the possible hazards of a substance as well as exposure of humans and the environment to it – proved to be slow, as were the subsequent introduction of risk management measures. The former system hampered research and innovation, causing the EU chemicals industry to lag behind its counterparts in the US and Japan in this regard.

All chemicals that were reported as being on the European Community market between 1 January 1971 and 18 September 1981 (listed in the European Inventory of Existing Commercial Chemical Substances – EINECS) were called “existing” chemicals. In 1981, they numbered more than 100.000 different substances. Chemicals introduced to the market after 1981 (more than 3 800) were termed “new” chemicals.

While new chemicals have to be tested before they are placed on the market, there were no such provisions for “existing” chemicals. Thus, although some information exists on the properties and uses of existing substances, there is generally a lack of sufficient information publicly available in order to assess and control these substances effectively.

Hence in 2001 the European Commission proposed to overhaul the current chemical control legislation and bring the measures for new and existing substances to the same standard, which necessitates a systematic review programme of existing substances. The new EU scheme will place a duty on manufacturers, importers and users of chemicals to assess the risks arising from their use and take any necessary risk management measures. The idea is to transfer the burden of proof from the regulators to industry for putting safe chemicals on the market.

## Objectives

In the Strategy for a Future Chemicals Policy, published in 2001, the Commission outlined the result of a review of the existing chemical management systems and laid the basis for a new strategy for ensuring a high level of chemicals safety and a competitive chemical industry through a system for the Registration, Evaluation and Authorisation of Chemicals – the REACH system.

The seven objectives that needed to be balanced within the overall framework of sustainable development were:

- Protection of human health and the environment
- Maintenance and enhancement of the competitiveness of the EU chemical industry
- Prevention of fragmentation of the internal market
- Increased transparency
- Integration with international efforts
- Promotion of non-animal testing
- Conformity with EU international obligations under the WTO

REACH represents the most major reform to the regulatory environment in which chemicals are used in the EU for over 25 years. The aim is “to improve the protection of human health and the environment through the better and earlier identification of the properties of chemical substances” as well as enhancing the “innovative capability and competitiveness of the EU chemicals industry”. REACH establishes an integrated system for registration, evaluation, and authorisation of chemicals. It extends the stringent testing requirements which have been imposed to chemicals introduced since 1981 also to those already in use.

The responsibility for providing sufficient information and taking effective risk management measures still lies principally with manufacturers or importers of chemicals. However, REACH introduces legal obligations further along the supply chain to anyone who uses chemicals and makes, or supplies the chemical products.

REACH applies to all substances (essentially chemicals) and preparations (mixtures of chemicals) which are imported, manufactured or used in volumes of 1 tonne *per annum* or more per producer or importer. There is no definitive list of chemicals that are covered by REACH as such but the existing EINECS and ELINCS lists cover around 100 000 chemicals. It is thought that around 30 000 of these will need to be put through the REACH process. Given the colossal volume, the process is staggered with the highest volume and most hazardous substances being dealt with first.

## Registration

Registration of information on the properties and uses are an integral part of the new system. The safety data requirements depend on the substance volume, and can be modified to take account of low exposure to humans or the environment. There will be a review programme for registration of “phase-in” (existing) substances, with priority given to higher tonnage substances and those with certain hazardous properties. Additional safety data are needed at higher tonnages, but for phase-in substances these could be provided later.

## Authorisation

Another key element of the scheme is EU authorisation of specific uses of very high concern substances triggered by certain hazardous properties. Although risk assessment is central to the REACH scheme, with a risk assessment as part of a Chemical Safety Report needed for registration of substances at above 10 tonnes *per annum*, authorisation decisions for substances with non-threshold hazardous properties, for which no safe dose or exposure can be assumed, decisions will be based on socio-economic benefits, taking into account the possibility of substitution of substances of lower risk.

## Information and testing

Manufacturers and importers of substances will be required to gather information on the properties of these if produced or imported in volumes over 1 tonne per year, and to submit this to a new European Chemicals Agency (ECHA).

This agency will assess whether this data is sufficient to show that relevant substance is safe to use. Failure to register will mean the substance cannot be manufactured, imported or used in the EU. The burden of proof is on industry to show that the risks associated with these chemicals are adequately and sufficiently controlled. Note that REACH also applies to chemicals released from products/articles.

There will be particularly stringent, use-specific authorisations required for chemicals that cause cancer, mutations or reproduction problems, or that accumulate in the body and in the environment. The intention is to encourage substitution of unsafe substances where possible.

When *de novo* testing will be required the tests should be performed according to the methods laid down in the Regulation (EC) No 440/2008. In the case of toxicological and ecotoxicological methods the tests and analyses shall be carried out in compliance with the principles of good laboratory practice provided for in Directive 2004/10/EC.

## Alternative tests

The promotion of alternatives to the animal testing of chemicals was an issue of prime concern to European Parliament in the process of REACH negotiations. The additional testing required under REACH will have great effects on laboratory animal use. At the same time, it will boost research into alternative test methods, based on the 3R principle (Replacement, Reduction and Refinement of laboratory animal use). To avoid duplication of animal testing, interested parties will have 45 days to state their views before each new plan for animal tests. Information on toxicity to human beings should if possible be discovered using means other than tests on vertebrate animals, through alternative methods such as in vitro procedures. These alternative methods must be validated by the Commission, once recognised by the agency (ECHA), or international institutions. The Commission will submit a report every three years on the use of alternative tests and, if necessary, bring forward fresh legislative proposals.

## How will REACH work?

All manufacturers and importers of chemicals must identify and manage risks linked to the substances they manufacture and market. For substances produced or imported in quantities of 1 tonne or more per year per company, manufacturers and importers need to demonstrate that they have appropriately done so by means of a registration dossier, which must be submitted to the European Chemicals Agency (ECHA).

The Agency may then check that the registration dossier complies with the Regulation and must evaluate testing proposals to ensure that the assessment of the chemical substances will not result in unnecessary testing, especially on animals.

Where appropriate, authorities may also select substances for a broader substance evaluation to further investigate substances of concern.

REACH also foresees an authorisation system aiming to ensure that substances of very high concern are properly controlled, and progressively replaced by suitable alternative substances or technologies where these are economically and technically viable. Where this is not possible, the use of substances may only be authorised where there is an overall benefit for society of using the substance.

In addition, EU authorities may impose restrictions on the manufacture, use or placing on the market of substances causing an unacceptable risk to human health or the environment.

The Member States authorities are responsible for enforcing REACH through inspections as well as penalties in case of non-compliance.

REACH was adopted in December 2006 with entry into force throughout the EU from 30 June 2007. As an EU regulation there is no transposition process to member state legislation as applies in the case of some other EU directives in the field. The timeline for REACH is complex depending on the volume in which a substance is used, how hazardous it is and how it is used. The most significant immediate deadline is 1 December 2008. Failure to register a substance already on the market (so called “phase-in” substances) by this date will mean it is illegal to import, manufacture or use it in the EU.

REACH replace the current legislation and place greater responsibility on industry. Manufacturers and importers will be required to gather comprehensive information on the properties of all substances produced or imported in quantities higher than 1 ton per year and to submit the necessary information to demonstrate their safe use in a registration dossier to the European Chemicals Agency. For the most dangerous substances, there will be an obligation for producers to submit a substitution plan to replace them with safer alternatives. Failure to register will mean the substance cannot be manufactured or imported into the EU market. The calendar for registration depends on the risk of the substance and the quantity produced. Currently about 30,000 substances are in the EU market in volumes above one tonne.

The regulation will enter into force progressively from June 2007, and the registration process will take 11 years to be completed. Consequently, all covered substances will have to be registered by 2018. A new European Chemicals Agency, based in Helsinki, will be responsible for the all activities required by REACH.
